# The Association of CHA_2_DS_2_-VASc Score and Blood Biomarkers with Ischemic Stroke Outcomes: The Belgrade Stroke Study

**DOI:** 10.1371/journal.pone.0106439

**Published:** 2014-09-03

**Authors:** Tatjana S. Potpara, Marija M. Polovina, Dijana Djikic, Jelena M. Marinkovic, Nikola Kocev, Gregory Y. H. Lip

**Affiliations:** 1 Faculty of Medicine, University of Belgrade, Belgrade, Serbia; 2 Cardiology Clinic, Clinical Center of Serbia, Belgrade, Serbia; 3 University Clinical Centre Gracanica, Kosovo, Serbia; 4 Institute for Medical Statistic and Informatic, University of Belgrade, Belgrade, Serbia; 5 University of Birmingham Centre for Cardiovascular Sciences, City Hospital, Birmingham, United Kingdom; University Hospital Medical Centre, Germany

## Abstract

**Background:**

Many blood biomarkers have a positive association with stroke outcome, but adding blood biomarkers to the National Institutes of Health Stroke Scale (NIHSS) did not significantly improve its discriminatory ability. We investigated the association of the CHA_2_DS_2_-VASc score with unfavourable functional outcome (defined as a 30-day modified Rankin Scale [mRS] ≥3) in patients presenting with acute ischemic stroke (AIS), and examined whether the addition of blood biomarkers (troponin I [TnI], fibrinogen, C-reactive protein [CRP]) affects the model discriminatory ability.

**Methods:**

We conducted an observational single-centre study of consecutive patients with AIS. All patients were admitted to hospital within 24 hours from the neurological symptoms onset.

**Results:**

Of 240 patients (mean age 70.0±8.9 years), unfavourable 30-day outcome occurred in 92 (38.3%). Patients with mRS≥3 were older and more likely to have atrial fibrillation or other comorbidities (all p<0.001). They had higher levels of CRP, fibrinogen, TnI and higher CHA_2_DS_2_-VASc and CHADS_2_ scores (all p<0.05). The adjusted CHA_2_DS_2_-VASc score had excellent predictive ability for poor stroke outcome (c-statistic 0.982;95%CI,0.964–1.000, p<0.001). Whilst CRP had the highest sensitivity (83.7%), cardiac TnI was the most specific (97.3%) for prediction of poor stroke outcome (cut-off: >0.09µg/L). Compared with each of these biomarkers, CHA_2_DS_2_-VASc score had significantly better predictive ability for poor stroke outcome (c-statistic for CRP, Fibrinogen and TnI was 0.853;95%CI,0.802–0.895, 0.848;95%CI,0.796–0.891, and 0.792;95%CI,0.736–0.842, all p<0.001, respectively, versus 0.932;95%CI,0.892–0.960, p<0.001 for the CHA_2_DS_2_-VASc, all p for the comparisons<0.01). There was no significant difference in the predictive ability of the CHA_2_DS_2_-VASc score vs. combinations of the CHA_2_DS_2_-VASc and TnI or TnI, fibrinogen and CRP (z statistic 0.369, p = 0.7119; integrated discrimination index 0.00801 and 0.00172, respectively, both p>0.05).

**Conclusions:**

The CHA_2_DS_2_-VASc score alone reliably predicts 30-day unfavourable outcome of stroke. Adding blood biomarkers to the CHA_2_DS_2_-VASc score did not significantly increase the predictive ability of the model.

## Introduction

The early prediction of death or disability following acute ischemic stroke (AIS) presently relies upon clinical variables such as age and stroke severity, as measured by the National Institutes of Health Stroke Scale (NIHSS) [1], [2]. These predictions are often broadly similar to the experienced stroke physicians clinical judgement [3], and continuous efforts are being made to improve the predictive ability of validated prognostic clinical variables by adding various biomarkers (whether blood, urine or imaging-based) to the original models based on clinical risk factors.

Many blood-based biomarkers have been extensively studied as potential predictors of poor stroke outcome. However, most of the associations were relatively weak and no single class of biomarkers had a stronger association than the others [4]. Nevertheless, the effect of *cardiac* biomarkers was consistent, and a number of studies found an increased mortality in stroke patients with elevated cardiac troponin I (TnI) [5]–[7] or troponin T (TnT) [8], [9]. Indeed, adding high-sensitivity TnT to several clinical variables including age and stroke severity resulted in incremental discrimination and reclassification of patients in one study [10], whilst another study showed that positive association of many biomarkers (including TnT) became statistically insignificant after adjustment for age and baseline NIHSS, and adding the N-Terminal pro-BNP or Interleukin-6 (the only statistically significant biomarkers after the adjustment) to age plus NIHSS made no significant difference to the model discriminatory ability [11].

A recent study showed that the CHADS_2_ and CHA_2_DS_2_-VASc scores, which were originally formulated for risk assessment of stroke in patients with atrial fibrillation (AF) [12], were good predictors of 5-year outcomes in non-AF patients with AIS [13]. The CHA_2_DS_2_-VASc score correlated well with stroke severity in AF patients [14], and was a multivariate predictor of 90-day stroke outcome, independently of baseline NIHSS values [15].

The aim of the present study was to investigate the association of the CHA_2_DS_2_-VASc score with poor *short-term* (30-day) functional outcome in patients with AIS, regardless of the heart rhythm, and to examine whether the addition of TnI affects the model discriminatory ability regarding the poor short-term outcome of AIS. We tested the hypothesis that the CHA_2_DS_2_-VASc score is significantly associated with poor short-term stroke outcome and that adding TnI improves the model predictive ability.

## Materials and Methods

### Patient selection and study design

An observational single-centre study of consecutive patients presenting with AIS who were admitted to hospital during 2010 was conducted in the University Clinical Centre Gracanica. All patients gave written informed consent, and the University Clinical Centre Gracanica review board approved the study.

All patients were admitted to hospital within 24 hours from the neurological symptoms onset. The diagnosis of AIS was established using the clinical evaluation and computed tomography (CT) of the brain within the first 24 hours of the event onset in all patients, and during hospitalization as needed. Patients with unclear timing of symptoms onset and those with haemorrhagic stroke or transient ischemic attack (TIA) were excluded (TIA was defined as a transient episode of neurological dysfunction caused by focal brain, spinal cord, or retinal ischemia, without acute infarction) [16]. Patients with a history of prior myocardial infarction (MI) or coronary artery disease (CAD) documented by coronary angiography, as well as patients with symptoms and electrocardiographic (ECG) signs suggestive of transient myocardial ischemia or an acute coronary syndrome (ACS) were excluded from this study in an attempt to avoid the confounding interplay of elevated cardiac troponin, possible acute MI and stroke regarding the 30-day functional stroke outcome.

All patients underwent a detailed history and physical examination, blood and urine testing, 12-channel ECG recording, chest radiography and transthoracic echocardiography (TTE). A 24-hour ECG Holter monitoring was performed in all patients within the first three days of hospitalization. Cardiac diseases and non-cardiac disorders were noted in the presence of a detailed medical record or a self-reported history of the disease, or when standard diagnostic criteria were fulfilled at diagnostic evaluation. Patients with advanced mitral or aortic valve disease awaiting surgical valve repair were excluded from the study.

### Blood biomarkers

Blood samples were taken upon admission to hospital and processed within 1 hour. In addition to routine biochemistry, C-reactive protein (CRP), cardiac TnI and D-dimer were measured in each patient. CRP was measured by latex-enhanced nephelometry with the use of high-sensitivity assays on the Behring Nephelometer II analyzer (Dade-Behring Diagnostics, Deerfield, IL) with a lower detection limit of 0.2 mg/L and interassay CVs of 5–9%. TnITnI and D-dimer were measured by ELFA (Enzyme Linked Fluorescent Assay) method (VIDAS TNI ULTRA and VIDAS D-Dimer Exclusion Assay, by BioMerieux Clinical Diagnostics), with a lower detection limit of 0.01µg/L and measurement range of 0.01–30µg/L (10% CV [Coefficient of Variation] point 0.11µg/L) for TnI, and a lower detection limit of ≤0.045µg/mL and measurement range of 0.045–10µg/mL (the clinical cut-off 0.5µg/mL) for D-dimer. In all patients, a creatinine clearance test was done on a 24-hour urine sample.

### Stroke and bleeding risk scores

All stroke and bleeding scores were calculated on admission to hospital, according to the clinical status prior to acute stroke onset. The CHADS_2_ score was calculated by giving 1 point each for congestive heart failure, hypertension, age >75 years and diabetes, and 2 points for prior stroke or TIA, the CHA_2_DS_2_-VASc by giving 1 point each for congestive heart failure/left ventricular systolic dysfunction (left ventricular ejection fraction ≤40%), hypertension, diabetes, peripheral vascular disease (including prior MI or complex aortic plaque), age 65–74 years and female gender, and 2 points for prior stroke or TIA and for age ≥75 years, and the HAS-BLED by giving 1 point each for hypertension, abnormal renal function, abnormal liver function, prior stroke, labile INRs (International Normalized Ratio), age >65 years, concomitant drug (aspirin or non-steroidal anti-inflammatory drugs) or alcohol use [17].

### Combined scores

Combined scores were formulated using the CHA_2_DS_2_-VASc score and TnI or CRP or fibrinogen or all three biomarkers, thus resulting in the CHA_2_DS_2_-VASc-T, CHA_2_DS_2_-VASc-CRP, CHA_2_DS_2_-VASc-F and CHA_2_DS_2_-VASc-CRPFT score, respectively.

### Short-term stroke outcomes

In-hospital mortality was defined as death from any cause during the index hospitalization. A 30-day mRS was calculated as a measure of functional neurological outcome of AIS; the scale is graded from 0 to 6, with 0 for patients without any symptom, 3–5 for increasing disability and 6 for death, and a mRS of ≥3 signifies an unfavourable stroke outcome [18].

None of the patients received thrombolytic therapy, since it was not available in our centre during the study period. Nevertheless, symptomatic haemorrhagic transformation (sHT) of AIS, defined as a rapid significant clinical deterioration temporally related to HT documented by CT-scan or autopsy, was regarded as a serious adverse event in the evolution of AIS [19], [20].

### Statistical analyses

Following a test of statistical normality, continuous variables are presented as mean (±SD), or with a skewed distribution as median with interquartile range (IQR, 25^th^ to 75^th^ quartile). Categorical variables are reported as counts with percentages. The Student t-test was used for comparison of continuous variables with normal distribution, and Mann-Whitney test for continuous variables with skewed distribution (Table 1). Differences in categorical variables were tested by Chi-square test. Since blood biomarkers' levels had skewed distribution, a logarithm transformation was used, and all logistic regression analyses were performed (and reported) in two ways: first, a biomarker was entered as a log transformed continuous independent variable and then, the analysis was performed with biomarker levels grouped to quartiles (the latter was done to facilitate a meaningful clinical interpretation of the results), Table 2 and 3. The CHADS_2_, CHA_2_DS_2_-VASc and HAS-BLED scores and all combined scores were entered as continuous variables in all models. Following unadjusted analyses (Table 4), the adjustment was done for each analysis as detailed in the Table 5 legend. Unfavourable 30-day functional outcome of AIS, including in-hospital death (i.e., a mRS of ≥3) was the dependent variable in all analyses.

**Table 1:** Baseline characteristic of patients with acute ischemic stroke according to favourable (mRS<3) or unfavourable 30-day functional stroke outcome (mRS≥3), including in-hospital death.

A. Baseline parameters	All patients	mRS<3	mRS≥3	P
	N = 240	148 (61.7)	92 (38.3)	
Age (years)	70.0±8.9	67.2±8.1	74.5±8.4	<0.001
Males	139 (57.9)	77 (52.0)	62 (67.4)	0.022
Body mass index	29.2±2.3	29.2±2.2	29.1±2.4	0.737
Systolic blood pressure (mmHg)	150 (140–180)	150 (140–170)	150 (115–190)	0.606
Diastolic blood pressure (mmHg)	90 (80–105)	90 (80–105)	90 (80–110)	0.151
Heart rate (beats per minute)	80 (75–90)	80 (75–90)	90 (75–95)	0.791
Current smoker	134 (55.8)	83 (56.1)	51 (55.4)	1.000
Excessive alcohol consumption	36 (15.0)	29 (19.6)	7 (7.6)	0.015
NYHA class (mean)	2.2±0.4	2.1±0.3	2.3±0.6	<0.001
NYHA class I-II	202 (84.2)	136 (91.9)	66 (71.8)	<0.001
NYHA class III-IV	38 (20.2)	12 (8.1)	28 (28.2)	<0.001
*Comorbidities:*				
Atrial fibrillation	88 (36.7)	35 (23.6)	53 (57.6)	<0.001
Hypertension	178 (74.2)	107 (72.3)	71 (77.2)	0.450
Diabetes mellitus	72 (30.0)	28 (18.9)	44 (47.8)	<0.001
Heart failure	18 (7.5)	3 (2.0)	15 (16.3)	<0.001
Chronic kidney disease	11 (4.6)	6 (4.1)	5 (5.4)	0.753
Prior stroke (any)/TIA	24 (10.0)	1 (0.7)	23 (25.0)	<0.001
Stroke and bleeding risk assessment scores*				
CHADS_2_ (mean)	1.86±1.1	1.30±0.64	2.77±1.07	<0.001
median (IQR)	2.0 (1.0–2.0)	1.0 (1.0–2.0)	3.0 (2.0–3.8)	
CHA_2_DS_2_-VASc (mean)	3.64±1.66	2.70±1.10	5.14±1.25	<0.001
median (IQR)	3.5 (3.0–5.0)	3.0 (2.0–3.0)	5.0 (4.0–6.0)	
HAS-BLED (mean)	2.45±1.07	2.08±0.90	3.04±1.06	<0.001
median (IQR)	2.0 (2.0–3.0)	2.0 (1.0–3.0)	3.0 (2.0–4.0)	
*Echocardiographic parameters:*				
Left atrial diameter (cm)	4.28±0.64	4.08±0.53	4.61±0.67	<0.001
Left atrial volume index	42.9±10.9	39.3±8.8	48.7±11.4	<0.001
emsp;Left ventricular ejection fraction	56.3±8.9	58.7±5.6	52.4±11.5	<0.001
Left ventricular end-systolic diameter (cm)	3.75±0.42	3.65±0.31	3.91±0.51	<0.001
Mitral annulus calcification	62 (25.8)	5 (3.4)	57 (62.0)	<0.001

B.Treatment at discharge				
Beta blockers	110 (45.8)	63 (42.6)	47 (51.1)	0.231
Calcium antagonists	149 (62.1)	92 (62.2)	57 (61.0)	1.000
Digoxin	30 (12.5)	10 (6.8)	20 (21.7)	0.001
Amiodarone	11 (4.6)	3 (2.0)	8 (8.7)	0.024
ACEIs	134 (55.8)	98 (66.2)	36 (39.1)	<0.001
ARBs	77 (32.1)	51 (34.5)	26 (28.3)	0.394
Diuretics	116 (48.3)	56 (37.8)	60 (65.2)	<0.001
Statins	68 (28.3)	48 (32.4)	20 (21.7)	0.079
Aspirin	154 (64.2)	90 (60.8)	64 (69.6)	0.213

Values are presented as n (%) or mean ± standard deviation or median with interquartile range (IQR).

mRS, modified Rankin Scale (patients who died were assigned a mRS of 6); NYHA, New York Heart Association; TIA, transient ischemic attack; CHADS_2_, congestive heart failure, hypertension, age ≥75 years, diabetes mellitus, prior stroke or TIA; CHA_2_DS_2_-VASc, congestive heart failure or left ventricular ejection fraction ≤40%, hypertension, age ≥75 years, diabetes mellitus, prior stroke or TIA, vascular disease (including myocardial infarction and peripheral artery disease), age >65 years, female gender; HAS-BLED, hypertension, abnormal renal or liver function, prior stroke, labile INRs (International Normalized Ratio), elderly (>65 years), concomitant drugs or alcohol use; MAC, mitral annulus calcification; ACEI, angiotensin converting enzyme inhibitor; ARB, angiotensin receptor blocker.

**Table 2:** Baseline blood biomarkers levels in patients with acute ischemic stroke according to favourable (mRS<3) or unfavourable 30-day functional stroke outcome, including in-hospital death (mRS≥3).

Blood biomarkers	All patients	mRS<3	mRS≥3	P
	N = 240	148 (61.7)	92 (38.3)	
**C-reactive protein**, mean	16.70±17.89	7.96±10.75	30.77±18.14	<0.001
C-reactive protein, median (IQR)	4.0 (3.0–32.5)	4.0 (3.0–4.0)	35.0 (16.0–44.0)	<0.001
Quartile 1: <3.0	18 (7.5)	16 (10.8)	2 (2.2)	
Quartile 2: 3.0–3.9	49 (20.4)	44 (29.7)	5 (5.4)	
Quartile 3: 4.0–32.4	113 (47.1)	78 (52.7)	35 (38.0)	
Quartile 4: ≥32.5	60 (25.0)	10 (6.8)	50 (54.3)	
**Fibrinogen**, mean	4.15±2.12	3.25±1.17	5.61±2.47	<0.001
Fibrinogen, median (IQR)	3.50 (2.90–5.00)	3.15 (2.50–3.60)	5.40 (3.90–6.80)	<0.001
Quartile 1: <2.9	57 (23.8)	51 (34.5)	6 (6.5)	
Quartile 2: 2.9–3.4	51 (21.2)	44 (29.7)	7 (7.6)	
Quartile 3: 3.5–4.9	68 (28.3)	44 (29.7)	24 (26.1)	
Quartile 4: ≥5.0	64 (26.7)	9 (6.1)	55 (59.8)	
**Cardiac TnI**, mean	0.217±0.453	0.06±0.10	0.468±0.649	<0.001
Cardiac TnI, median (IQR)	0.06 (0.03–0.09)	0.04 (0.02–0.07)	0.20 (0.05–0.65)	<0.001
Quartile 1: <0.03	47 (19.6)	42 (28.4)	5 (5.4)	
Quartile 2: 0.03–0.059	70 (29.2)	51 (34.5)	19 (20.7)	
Quartile 3: 0.06–0.089	49 (20.4)	36 (24.3)	13 (14.1)	
Quartile 4: ≥0.09	74 (30.8)	19 (12.8)	55 (59.8)	
**D-dimer**, mean	0.61±0.85	0.45±0.68	0.87±1.02	<0.001
D-dimer, median (IQR)	0.24 (0.18–0.80)	0.22 (0. 17–0.32)	0.50 (0.19–1.24)	<0.001
Quartile 1: <0.180	56 (23.3)	41 (27.7)	15 (16.3)	
Quartile 2: 0.180–0.234	64 (26.7)	41 (27.7)	23 (25.0)	
Quartile 3: 0.235–0.799	58 (24.2)	44 (29.7)	14 (15.2)	
Quartile 4: ≥0.800	62 (25.8)	22 (14.9)	40 (43.5)	
**WBC**, mean	8.21±1.68	7.99±1.76	8.51±1.47	0.008
WBC, median (IQR)	8.1 (7.0–9.0)	7.8 (6.9–9.0)	8.80 (7.70–9.68)	0.072
Quartile 1: <7.0	50 (20.8)	38 (25.7)	12 (13.0)	
Quartile 2: 7.0–8.0	69 (28.7)	43 (29.1)	26 (28.3)	
Quartile 3: 8.1–8.9	38 (15.8)	23 (15.5)	15 (16.3)	
Quartile 4: ≥9.0	83 (34.6)	44 (29.7)	39 (47.0)	
**CrCl**, mean	55.35±7.60	56.36±7.35	53.73±7.77	0.009
CrCl, median (IQR)	56.00 (51.00–60.00)	56.00 (53.25–60.00)	55.00 (48.25–59.00)	0.020
Quartile 1: <51	58 (24.2)	27 (18.2)	31 (33.7)	
Quartile 2: 51–55	61 (25.4)	36 (24.3)	25 (27.2)	
Quartile 3: 56–59	52 (21.7)	38 (25.7)	14 (15.2)	
Quartile 4: ≥60	69 (28.7)	47 (31.8)	22 (23.9)	
**Total cholesterol**, mean	5.71±1.17	5.57±1.09	5.91±1.26	0.025
Total cholesterol, median (IQR)	5.35 (5.00–6.50)	5.20 (5.00–6.00)	6.00 (5.00–6.80)	
HDL, mean	1.04±0.46	1.02±0.31	1.07±0.63	0.416
HDL, median (IQR)	1.00 (0.90–1.00)	1.00 (0.93–1.05)	1.00 (0.90–1.00)	
LDL, mean	3.95±1.17	3.83±1.06	4.14±1.31	0.043
LDL, median (IQR)	3.80 (3.20–4.70)	3.60 (3.20–4.36)	4.40 (3.30–4.98)	
**Hematocrit**, mean	0.41±0.07	0.42±0.06	0.40±0.07	0.040
Haematocrit, median (IQR)	0.41 (0.38–0.45)	0.42 (0.39–0.45)	0.40 (0.34–0.46)	
**Haemoglobin**, mean	134.13±18.71	135.03±19.63	132.68±17.13	0.084
Haemoglobin, median (IQR)	135 (127–145.75)	136.50 (129–146)	134 (125.50–145)	

Values are presented as n (%) or mean ± standard deviation or median with interquartile range (IQR). C-reactive protein is given in mg/L, fibrinogen in g/L, TnI in μg/L and D-dimer in μg/mL.

mRS, modified Rankin Scale (patients who died were assigned a mRS of 6); WBC, white blood cell count; CrCl, creatinine clearance; HDL, high density lipoprotein; LDL, low density lipoprotein.

**Table 3:** Crude associations of biomarkers and the CHADS_2_, CHA_2_DS_2_-VASc and HAS-BLED score with unfavourable 30-day outcome of ischemic stroke, predictive ability of each biomarker or score, and pairwise comparisons of each biomarker and stroke risk score predictive ability.

					Predictive ability			Pairwise comparison	
		OR	95%CI	P	c-statistic	95%CI	P	z-statistic	P
	Biomarker								
1	CRP	3.9	2.9–5.3	<0.001	0.853	0.802–0.895	<0.001	0.335 (1 vs. 2)	0.7390
	quartiles	5.8	3.5–9.5	<0.001	0.795	0.738–0.844	<0.001	1.678 (1 vs. 3)	0.0934
								4.616 (1 vs. 4)	<0.001
								5.641 (1 vs. 5)	<0.001
								6.081 (1 vs. 6)	<0.001
2	Fibrinogen	61.7	21.1–180.7	<0.001	0.848	0.796–0.891	<0.001	1.536 (2 vs. 3)	0.1246
	quartiles	4.2	1.9–6.0)	<0.001	0.835	0.781–0.879	<0.001	4.312 (2 vs. 4)	<0.001
								5.530 (2 vs. 5)	<0.001
								5.549 (2 vs. 6)	<0.001
3	TnI	3.2	2.3–4.4	<0.001	0.792	0.736–0.842	<0.001	3.249 (3 vs. 4)	0.0012
	quartiles	2.7	2.0–3.7	<0.001	0.767	0.705–0.830	<0.001	3.441 (3 vs. 5)	0.0006
								3.847 (3 vs. 6)	0.0001
4	D-dimer	1.9	1.4–2.5	<0.001	0.642	0.578–0.703	<0.001	0.371 (4 vs. 5)	0.7109
	quartiles	1.6	1.2–2.0	<0.001	0.636	0.572–0.697	<0.001	0.626 (4 vs. 6)	0.5313
5	WBC	1.2	1.1–1.5	0.010	0.622	0.558–0.684	0.001	0.236 (5 vs. 6)	0.8134
	quartiles	1.3	1.1–1.7	<0.001	0.593	0.528–0.656	0.009		
6	CrCl	0.09	0.04–0.59	0.012	0.610	0.545–0.672	0.004		
	quartiles	0.7	0.5–0.7	0.007	0.601	0.536–0.664	0.006		
	**Stroke risk score**								
7	CHA_2_DS_2_-VASc	7.9	4.6–13.4	<0.001	0.932	0.892–0.960	<0.001	4.265 (7 vs. 8)	<0.001
8	CHADS_2_	10.8	5.6–21.0	<0.001	0.872	0.823–0.912	<0.001	3.834 (8 vs. 9)	<0.001
9	HAS-BLED	2.8	2.0–3.8	<0.001	0.750	0.690–0.804	<0.001	5.674 (7 vs. 9)	<0.001
								**z-statistic**	**P**
	**CHA_2_DS_2_-**				CHA_2_DS_2_-VASc vs. CRP			2.963	0.0030
	**VASc score vs. biomarkers**				CHA_2_DS_2_-VASc vs. Fibrinogen			2.961	0.0031
					CHA_2_DS_2_-VASc vs. TnI			3.977	0.0001

OR, Odds Ratio; CI, Confidence Interval.

Only biomarkers with p<0.01 regarding unfavourable 30-day functional stroke outcome are shown.

CRP, C-reactive protein; WBC, white blood cell count; CrCl, creatinine clearance; CHA_2_DS_2_-VASc, congestive heart failure or left ventricular ejection fraction ≤40%, hypertension, age ≥75 years, diabetes mellitus, prior stroke or TIA, vascular disease (including myocardial infarction and peripheral artery disease), age >65 years, female gender (1 point each, age ≥75 years and prior stroke/TIA 2 points each); CHADS_2_, congestive heart failure, hypertension, age ≥75 years, diabetes mellitus, prior stroke or TIA (1 point each, prior stroke/TIA 2 points); HAS-BLED, hypertension, abnormal renal or liver function, prior stroke, labile INRs (International Normalized Ratio), elderly (>65 years), concomitant drugs or alcohol use.

**Table 4:** Crude associations of the CHA_2_DS_2_-VASc score and combinations of the score plus C-reactive protein, fibrinogen or TnI, or the CHA_2_DS_2_-VASc score plus all three biomarkers with unfavourable 30-day functional outcome of ischemic stroke and pairwise comparisons of the scores.

	Score	OR	95%CI	P	c-statistic	95%CI	P	z-statistic	P
1	CHA_2_DS_2_-VASc	7.9	4.6–13.4	<0.001	0.932	0.892–0.960	<0.001	2.268 (2 vs. 1)	0.0233
2	CHA_2_DS_2_-VASc-CRP	5.9	3.8–9.3	<0.001	0.948	0.911–0.972	<0.001	2.020 (3 vs. 1)	0.0434
3	CHA_2_DS_2_-VASc-F	6.0	3.8–9.5	<0.001	0.946	0.909–0.971	<0.001	3.274 (4 vs. 1)	0.0011
4	CHA_2_DS_2_-VASc-T	10.0	5.3–18.9	<0.001	0.955	0.921–0.978	<0.001	1.947 (5 vs. 1)	0.0515
5	CHA_2_DS_2_-VASc-CRFT	3.6	2.6–4.8	<0.001	0.953	0.917–0.976	<0.001	1.514 (3 vs. 2)	0.1299
								1.242 (4 vs. 2)	0.2144
								0.995 (5 vs. 2)	0.3195
								1.433 (4 vs. 3)	0.1517
								1.267 (5 vs. 3)	0.2053
								0.427 (5 vs. 4)	0.6692

CHA_2_DS_2_-VASc, congestive heart failure or left ventricular ejection fraction ≤40%, hypertension, age ≥75 years, diabetes mellitus, prior stroke or TIA, vascular disease (including myocardial infarction and peripheral artery disease), age >65 years, female gender (1 point each, age ≥75 years and prior stroke/TIA 2 points each); CHA_2_DS_2_-VASc-CRP, 1 additional point if C-reactive protein was >4 mg/L; CHA_2_DS_2_-VASc-F, 1 additional point if fibrinogen was >3.7 g/L; CHA_2_DS_2_-VASc-T, 1 additional point if TnI was >0.09µg/L; CHA_2_DS_2_-VASc-CRPFT, 1 additional point for each of three biomarkers if above the correspondent cut-off level.

**Table 5:** The adjusted associations of biomarkers, ‘classic’ scores and ‘composed’ scores with unfavourable 30-day functional outcome of ischemic stroke, with model discriminatory ability and goodness of model fit, and pairwise comparison of each model.

		Adjusted analysis		Model discriminatory ability		Model fit	Pairwise comparisons of the ROC curves		
	Variable	OR (95% CI)	P	c-statistic (95% CI)	P	Hosmer-Lemeshow test (HL)	z-statistic	P	IDI
						Nagelkerke R square (NR^2^)			P
									0.00801
1	TnI (quartiles)	1.8 (1.1–3.2)	0.036	0.957 (0.931–0.983)	<0.001	HL 0.036; NR^2^ 0.786	2.576 (3 vs. 1)	0.0100	(3 vs. 4)
2	Fibrinogen (quartiles)	2.2 (1.8–4.1)	0.013	0.957 (0.931–0.983)	<0.001	HL 0.036; NR^2^ 0.786	2.576 (3 vs. 2)	0.0100	P = 0.15460
3	CHA_2_DS_2_-VASc	14.1 (3.4–59.5)	<0.001	0.982 (0.964–1.000)	<0.001	HL <0.001; NR^2^ 0.870	0.369 (4,5 vs. 3)	0.7119	0.00172
4	CHA_2_DS_2_-VASc-T	22.5 (3.1–162.9)	0.002	0.983 (0.963–1.000)	<0.001	HL <0.001; NR^2^ 0.877	2.588 (4 vs. 1,2)	0.0097	(3 vs. 5)
5	CHA_2_DS_2_-VASc-CRPFT	7.7 (1.8–32.7)	0.006	0.983 (0.963–1.000)	<0.001	HL <0.001; NR^2^ 0.877	2.588 (5 vs. 1,2)	0.0097	P = 0.61428

OR, Odds Ratio; CI, Confidence Interval; ROC, Receiver Operating Characteristic; IDI – integrated discrimination index.

Only biomarkers or scores with significant association with unfavourable 30-day functional stroke outcome following adjustment are shown.

Cardiac TnI and fibrinogen adjusted for other biomarkers (white blood cell count, haemoglobin, haematocrit, C-reactive protein, D-dimer, creatinine clearance, total cholesterol, high-density lipoprotein and low-density lipoprotein), age, gender, body mass index, smoking status, alcohol consumption, NYHA class, atrial fibrillation, history of hypertension, heart failure, diabetes mellitus, chronic kidney disease, prior stroke/TIA, left atrial volume, left ventricular ejection fraction, the presence of mitral annular calcification and symptomatic haemorrhagic transformation of ischemic stroke.

The CHA_2_DS_2_-VASc score adjusted for CHADS_2_ and HAS-BLED score, all biomarkers, gender, body mass index, smoking status, alcohol consumption, NYHA class, atrial fibrillation, chronic kidney disease, left atrial volume, left ventricular ejection fraction, the presence of mitral annular calcification and symptomatic haemorrhagic transformation of ischemic stroke.

The CHA_2_DS_2_-VASc-T and CHA_2_DS_2_-VASc-CRPFT scores adjusted for all blood biomarkers, gender, body mass index, smoking status, alcohol consumption, NYHA class, atrial fibrillation, chronic kidney disease, left atrial volume, left ventricular ejection fraction, the presence of mitral annulus calcification and symptomatic haemorrhagic transformation of ischemic stroke.

The *c-*statistic, a measure of the area under the receiver-operator characteristic (ROC) curve, was used to quantify the predictive validity of each biomarker, the CHADS_2_, CHA_2_DS_2_-VASc and HAS-BLED and the combined scores, and tested the hypothesis that these models performed significantly better than chance (indicated by a *c-*statistic ≥0.5). In addition to model discriminatory ability (the ROC curve analysis), model calibration of each adjusted model was tested by the Hosmer-Lemeshow goodness-of-fit test, which assesses whether the observed event rates match expected event rates in subgroups of the model population. Explanatory power was tested using the pseudo- *R* 2 statistic according to the “Nagelkerke R^2^ ” to assess the degree to which the model explained the variance of the binary outcome. Pairwise comparisons of the ROC curves were performed using the approach of DeLong, DeLong and Clarke-Pearson [21] and the MedCalc statistical software – version 12.7.0.0. Finally, using the Stata 11 software, we calculated the integrated discrimination index (IDI), which is equivalent to the difference in discrimination slopes [22]. All other statistical analyses were performed using SPSS 20.0 software package (SPSS Inc., Chicago, Illinois). A value of P <0.05 was considered statistically significant in all analyses.

## Results

Of 273 patients with AIS, 33 were excluded from this study because of the history of CAD or an acute MI during the hospitalization for AIS. These patients were not significantly older as compared to those without clinically evident CAD (73.2±10.1 vs. 70.0±8.9 years, respectively, p = 0.064), but they had higher CHADS_2_ and CHA_2_DS_2_-VASc scores (2.82±1.21 vs. 1.86±1.10 and 4.91±1.65 vs. 3.64±1.66, respectively, both p<0.001) and mean NYHA class (2.6±0.8 vs. 2.2±0.4, p<0.001), lower CrCl (50.9±9.3 vs. 55.4±7.6, p = 0.002) and higher CRP levels (27.3±16.6±16.7±17.9, p = 0.001), whilst there was no significant difference in the mean TnI levels at baseline (0.32±0.35 vs. 0.22±0.45, p = 0.266) or the prevalence of AF (18 [54.5%] vs. 88 [36.7%], p = 0.057). An unfavourable 30-day stroke outcome occurred more frequently in the CAD patients (28 [84.8] vs. 92 [38.3]%, p<0.001).

Of 240 patients included in this study (Table 1), unfavourable 30-day functional stroke outcome occurred in 92 patients (38.3%), including in-hospital death in 21 patients (8.8%). Patients with mRS≥3 were older, more often males and more likely to have AF, diabetes mellitus, HF, prior stroke/TIA, mitral annulus calcification, larger left atrial size or lower left ventricular ejection fraction (all p<0.001). They were more often prescribed diuretics and digoxin, and less frequently received angiotensin-converting enzyme inhibitors (all p<0.05).

### Blood biomarkers

**Figure 1:**
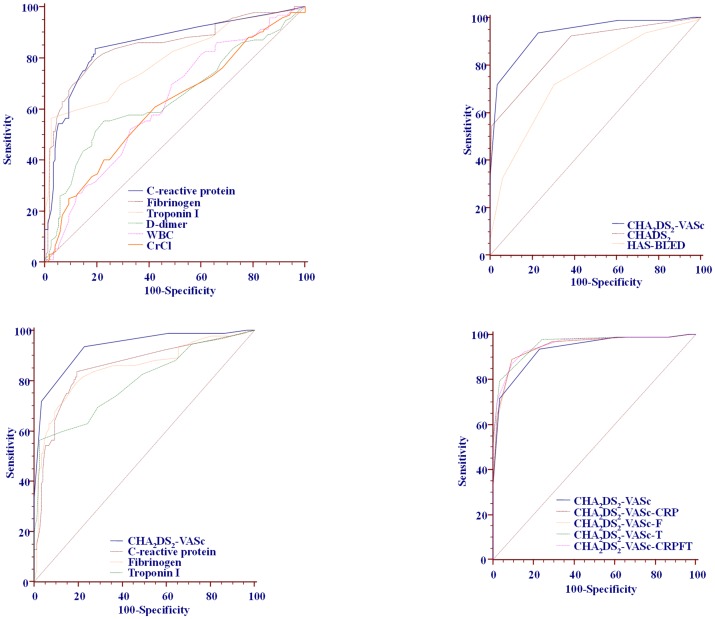
Receiver-operator characteristic analyses of the biomarkers and stroke risk scores predictive ability for unfavourable 30-day outcome of ischemic stroke and pairwise comparisons (Table 2).

Patients with 30-day mRS≥3 had higher levels of CRP, fibrinogen, TnI, D-dimer, total cholesterol and LDL, higher white blood cell count, and lower hematocrit and CrCl (all p<0.05, Table 2]. Significant associations of blood biomarkers with unfavourable 30-day stroke outcome, predictive ability of each biomarker and pairwise comparisons of biomarker predictive abilities are shown in Table 3 and Figure 1 (upper left panel). CRP, fibrinogen and TnI had significantly better predictive ability compared with D-dimer, CrCl or total cholesterol (all p<0.001), whilst there were no significant differences among the first three biomarkers (Table 3 and Figure 1).

**Figure 2:**
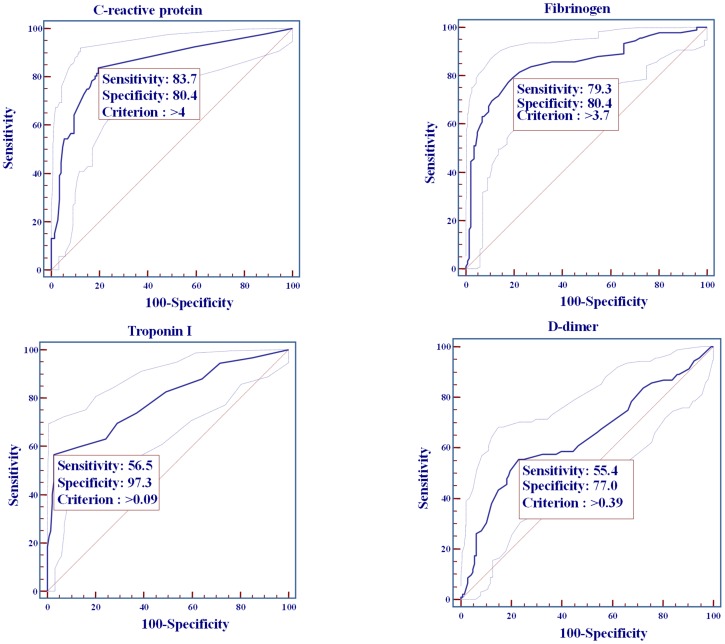
C-reactive protein, fibrinogen, TnI and D-dimer sensitivity, specificity and associated criterion for unfavourable 30-day outcome of ischemic stroke.

Sensitivity, specificity and cut-off values for cardiac CRP, fibrinogen, TnI and D-dimer are shown in Figure 2. Whilst CRP had the highest sensitivity (83.7%), cardiac TnI was the most specific (97.3%) for prediction of unfavourable short-term stroke outcome, with cut-off value of >0.09µg/L (Figure 2).

### CHA_2_DS_2_-VASc, CHADS_2_ and HAS-BLED scores

Patients with unfavourable short-term functional stroke outcome had higher values of all three scores, compared with patients with mRS<3. The crude association of CHA_2_DS_2_-VASc, CHADS_2_ and HAS-BLED scores with unfavourable 30-day outcome, predictive ability of each score and pairwise comparison of the scores predictive abilities are shown in Table 3 and Figure 1 (upper right panel). The CHA_2_DS_2_-VASc score had significantly better predictive ability for unfavourable short-term stroke outcome than the CHADS_2_ or HAS-BLED score (both p<0.001) [Table 3 and Figure 1]. Within the CHA_2_DS_2_-VASc score, age, diabetes mellitus and prior stroke or TIA were significantly associated with unfavourable 30-day outcome (Odds Ratio [OR] 1.2; 95% Confidence Interval [CI] 1.1–1.2, OR 6.3; 95%CI 2.8–14.0 and OR 47.7; 95%CI 5.2–439.0, respectively, all p<0.001).

The CHA_2_DS_2_-VASc score had better sensitivity and specificity than the CHADS_2_ or HAS-BLED score (93.5% vs. 92.4% vs. 71.7% and 77.0% vs. 61.5% vs. 69.6%, respectively, all p<0.05) for unfavourable short-term functional outcome of AIS.

### CHA_2_DS_2_VASc-score versus CRP, fibrinogen or cardiac TnI

Pairwise comparisons of predictive abilities of the CHA_2_DS_2_-VASc score versus CRP, fibrinogen or TnI are shown in Table 3 and Figure 1 (lower left panel). Compared with each of these biomarkers, CHA_2_DS_2_-VASc score had significantly better predictive ability for unfavourable 30-day functional stroke outcome (all p<0.01).

### Combined scores (CHA_2_DS_2_VASc plus CRP or fibrinogen or TnI and CHA_2_DS_2_VASc plus all three biomarkers)

As above, the cut-off values for increased risk of unfavourable stroke outcome in our cohort were determined for each of three biomarkers, and 1 point was added to the CHA_2_DS_2_-VASc score if the level of a given biomarker in a given patient exceeded the cut-off value.

On the unadjusted analyses, all combined scores (Table 4) were significantly associated with unfavourable 30-day functional stroke outcome (all p<0.001), and the combination of CHA_2_DS_2_-VASc with TnI had the best predictive ability (c-statistic 0.955; 95%CI,0.921–0.978, p<0.001). All unadjusted combined scores were slightly better than unadjusted CHA_2_DS_2_-VASc alone (all p≤0.05). The combination of CHA_2_DS_2_-VASc with all three biomarkers (CHA_2_DS_2_-VASc-CRPFT) had similar predictive ability as each of the combinations of CHA_2_DS_2_-VASc score with a single biomarker (all p≥0.05) [Table 4 and Figure 1 (lower right panel)].

**Figure 3:**
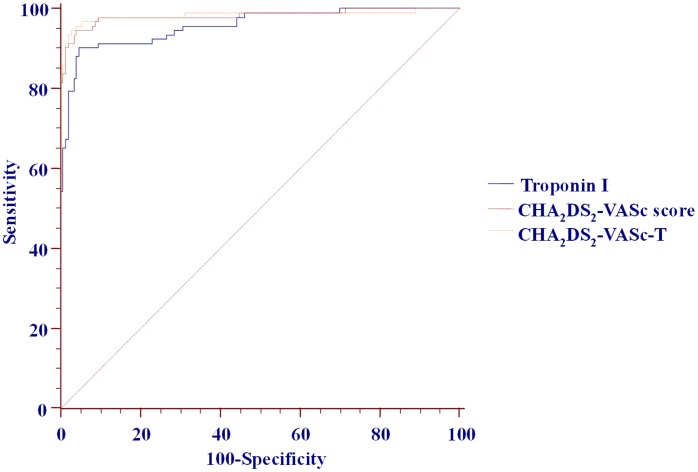
Pairwise comparison of Receiver-operator characteristic of fully adjusted TnI, ‘classic’ and composed scores for unfavourable 30-day functional outcome of ischemic stroke.

Following adjustment (see Table 5 legend), only TnI, fibrinogen, CHA_2_DS_2_-VASc, CHA_2_DS_2_-VASc-T and CHA_2_DS_2_-VASc-CRPFT score remained significantly associated with unfavourable 30-day stroke outcome (Table 5). The pairwise comparisons of their predictive abilities are shown in Table 5 and Figure 3. Adjusted CHA_2_DS_2_-VASc and CHA_2_DS_2_-VASc-T (or CHA_2_DS_2_-VASc-CRPFT) score models had significantly better predictive ability than TnI alone, whilst there was no significant difference between adjusted CHA_2_DS_2_-VASc vs. CHA_2_DS_2_-VASc-T score (or the CHA_2_DS_2_-VASc-CRPFT) models (all p>0.05), with statistically insignificant discrimination improvement when adding TnI only or CRP plus fibrinogen plus TnI to the ‘classic’ CHA_2_DS_2_-VASc score (Table 5).

### Analyses including patients with clinically evident CAD

Analyses of the whole AIS cohort (n = 273) including patients with clinically evident CAD (n = 33) yielded essentially identical results. Patients with unfavourable short-term functional stroke outcome had higher values of all three scores, compared with patients with mRS<3 (mean CHADS_2_ of 2.83±1.10 vs. 1.31±0.64, mean CHA_2_DS_2_-VASc of 5.17±1.31 vs. 2.71±1.10 and mean HAS-BLED score of 3.06±1.06 vs. 2.08±0.91, respectively, all p<0.001).

All 3 scores were significantly associated with unfavourable 30-day stroke outcome on the univariate analyses (CHADS_2_: OR 11.9, 95%CI 6.3–22.4; CHA_2_DS_2_-VASc: OR 7.6, 95%CI 4.7–12.2; HAS-BLED: OR 2.8, 95%CI 2.1–3.8, all p<0.001), whilst only the CHA_2_DS_2_-VASc was significantly associated with unfavourable 30-day stroke outcome in the multivariable analysis including all 3 scores (OR 7.6, 95%CI 4.7–12.2). All 3 scores had a good predictive ability for unfavourable short-term stroke outcome (CHADS_2_: c-statistic 0.882, 95%CI 0.841–0.922; CHA_2_DS_2_-VASc: c-statistic 0.932, 95%CI 0.91–0.962; HAS-BLED: c-statistic 0.754, 95%CI 0.696–0.812, all p<0.001), but the CHA_2_DS_2_-VASc score had significantly better predictive ability compared with the CHADS_2_ or HAS-BLED score (z-statistic 4.256 and 5.648, respectively, both p<0.001). Within the CHA_2_DS_2_-VASc score, age, diabetes mellitus, prior stroke or TIA and CAD were significantly associated with unfavourable short-term stroke outcome (OR [95%CI]: 1.2 [1.1–1.2], 5.3 [2.5–11.1], 43.9 [5.0–384.3] and 8.5 [2.3–31.5], respectively, all p ≤0.001). Other analyses with the biomarkers and combined scores also yielded essentially identical results as shown for the AIS cohort without clinically overt CAD (data not shown).

## Discussion

In the present study, we show that the CHA_2_DS_2_-VASc score reliably predicts a short-term, 30-day unfavourable outcome of AIS (regardless of the presence or absence of AF), and that adding the high-sensitivity TnI (or fibrinogen or CRP, or all three biomarkers) to CHA_2_DS_2_-VASc score does not further improve the prediction. These findings could be of clinical relevance, as the pre-stroke CHA_2_DS_2_-VASc score can be easily calculated in most circumstances, thus offering a simplified, quick and reliable initial risk assessment in the acute stroke setting.

The CHA_2_DS_2_-VASc score has been originally developed to refine the assessment of stroke risk in patients with non-valvular AF [12], and was subsequently validated in a number of AF cohorts [23]–[27]. Recently, it was tested in a non-AF cohort and was comparably successful for stroke prediction in that cohort as in AF patients [28]. In addition, many recent studies showed that the CHA_2_DS_2_-VASc score was associated with poor clinical outcomes, including mortality, in various cohorts of cardiovascular patients [29]. Furthermore, the CHA_2_DS_2_-VASc score has been shown to correlate well with stroke severity in AF patients [14], and to be a multivariable predictor of 90-day stroke outcome, independently of baseline NIHSS values [15]. A recent study found that the CHADS_2_ and CHA_2_DS_2_-VASc scores also were good predictors of 5-year outcomes in non-AF patients with AIS [13]. However, the association of the CHADS_2_ and CHA_2_DS_2_-VASc scores with 30-day outcome of AIS has not been previously investigated prospectively [30], [31].

Our findings suggest that the CHA_2_DS_2_-VASc score reliably predicts poor 30-day stroke outcome, with significantly better predictive ability compared to the CHADS_2_ score. Indeed, on the logistic regression analysis which accounted for blood biomarkers, the presence of AF and a number of clinical/echocardiographic parameters, the model with CHA_2_DS_2_-VASc score had an excellent predictive ability for poor short-term stroke outcome (c-statistic of 0.982). Indeed, even the unadjusted CHA_2_DS_2_-VASc score predictive ability was very good, with c-statistic of 0.932.

Increasing body of evidence shows that a number of blood biomarkers are significantly associated with various clinical events [4], [11], [32], [33], [34], [35]. For example, a recent large biomarker substudy of the RE-LY trial (Randomized Evaluation of Long-Term Anticoagulation Therapy) showed that elevated TnI and NT-proBNP were independently related to increased risks of stroke and mortality and significantly improved risk prediction in AF patients beyond currently used clinical tools (i.e., the CHADS_2_ and CHA_2_DS_2_-VASc scores) [36]. However, the substudy had not investigated the association of biomarkers with stroke outcomes in patients who suffered AIS during follow-up. A recent systematic review of diagnostic and prognostic role of blood biomarkers in AIS highlighted blood glucose and fibrinogen levels as the most consistent predictors of poor functional outcome of stroke [33]. Nonetheless, studies on biomarkers generally suffer from several shortcomings, including relatively small number of patients, variable analytical techniques and interpretation of results (which may limit the reproducibility), different study population risk profiles, etc. Indeed, a recent study of 270 patients with AIS or TIA, which investigated the prognostic role of 18 blood biomarkers (including glucose, fibrinogen, troponin T, CRP, D-dimer, leukocytes, etc.) for 90-day functional outcome, found that on adjusted analyses only Interleukin-6 and NT-proBNP were significantly related to poor 90-day outcome, but with no significant improvement in predictive ability when added to age plus NIHSS model [11].

In our study, adjusted TnI and fibrinogen were significantly related to 30-day poor functional outcome of stroke. However, upon adjustment for other biomarkers and clinical/echocardiographic variables, the CHA_2_DS_2_-VASc-F score (i.e., the ‘classic’ score plus fibrinogen) was no longer significantly associated with 30-day poor outcome, in contrast to the CHA_2_DS_2_-VASc-T score (i.e., the ‘classic’ score plus TnI). Cardiac troponin (a protein involved in the heart muscle contraction) is a well established indicator of myocardial damage, and elevations in troponin levels have been observed in patients with acute MI, stable CAD, HF, renal failure, pulmonary embolism, and even in elderly apparently healthy individuals [32], [33], [37]–[42]. In addition, it has been shown that more than half AF patients have detectable levels of TnI [36]. Importantly, troponin has been uniformly associated with worse outcomes and increased mortality in all these cohorts, independent of traditional major coronary risk factors [32], [33], [36]–[39].

Increased troponin T levels have been reported in 5–34% of patients with AIS [43], and the elevation of troponin T was associated with stroke severity, insular cortex lesions, short- and long-term clinical outcome and increased risk of mortality, thus indicating prognostic significance of increased troponin T in AIS [43]–[47]. TnI increase in the AIS patients may be caused by the coincident ACS with myocardial necrosis or by a neurogenic cardiac damage due to autonomic imbalance in the acute stroke setting [48], [49]. It is not always possible to elucidate the exact cause of elevated TnI an individual patient with AIS, and an ongoing study will try to prospectively determine the frequency and possible etiology of troponin elevation in a large cohort of AIS patients, using coronary angiography [49]. In our study, all patients with known CAD and those with clinical, electrocardiographic and/or echocardiographic findings suggesting myocardial ischemia or acute MI at baseline (or during the subsequent 4 weeks) were excluded, in an effort to eliminate the impact of possible acute MI on short-term outcome of acute stroke (nonetheless, we are aware of the possibility that some of our patients could still suffer an unrecognized MI).

Overall, our findings suggest that regardless of the significant association of high-sensitivity TnI and fibrinogen with short-term functional outcome of AIS, these biomarkers do not significantly improve the excellent predictive ability of the CHA_2_DS_2_-VASc score alone. Hence, a routine measurement of cardiac TnI in patients presenting with AIS does not seem justified unless an ACS is suspected. Given the relatively small size of our study, these findings (and their potential implications for treatment decisions) need further evaluation in larger cohorts with acute ischemic stroke.

### Study limitations

This was a single centre study performed in a university hospital and our findings might not be fully reflective of a high volume centre setting [50], although a 8.8% in-hospital mortality in our study is comparable to other reports [50], [51]. Nonetheless, our findings should be interpreted with some caution, given the relatively small number of participants in our study. Since the baseline NIHSS was not available for majority of our patients, the results were not adjusted for stroke severity. The NIHSS has been shown to be an important predictor of mortality in AIS [1], [52], but less than 50% of hospitals participating in the Get With The Guidelines – Stroke regularly reported patient NIHSS scores in a recent study [48], and the CHA_2_DS_2_-VASc score has been shown previously to be a good predictor of stroke outcome in a model which included NIHSS [15]. In addition, although all patients in our study underwent a 24-hour Holter monitoring, there is still a possibility of undiagnosed AF, which could affect the outcomes. Finally, the observational design of our study does not exclude a possibility of patient selection bias and residual confounding, although we have prospectively included consecutive patients presenting with AIS during one calendar year.

## Conclusion

High-sensitivity TnI, fibrinogen and the CHA_2_DS_2_-VASc score predict 30-day unfavourable functional outcome of AIS regardless of the presence or absence of AF and other cardiovascular comorbidities or risk factors. The CHA_2_DS_2_-VASc score has better predictive ability for stroke outcome than TnI or fibrinogen, and adding these biomarkers to the CHA_2_DS_2_-VASc score does not further improve the prediction of poor 30-day stroke outcome in the absence of clinically evident CAD. The use of CHA_2_DS_2_-VASc score could facilitate a simplified, quick and reliable initial risk assessment of patients presenting with AIS.
